# Mamba6mA: a Mamba-based DNA N6-methyladenine site prediction model

**DOI:** 10.1093/bioinformatics/btag060

**Published:** 2026-02-05

**Authors:** Qi Zhao, Zhen Zhang, Tingwei Chen, Qian Mao, Haoxuan Shi, Jingjing Chen, Zheng Zhao, Xiaoya Fan

**Affiliations:** Faculty of Information, Liaoning University, Shenyang, Liaoning, 110036, China; College of Medicine and Biological Information Engineering, Northeastern University, Shenyang, Liaoning, 110000, China; College of Medicine and Biological Information Engineering, Northeastern University, Shenyang, Liaoning, 110000, China; Faculty of Information, Liaoning University, Shenyang, Liaoning, 110036, China; College of Light Industry, Liaoning University, Shenyang, Liaoning, 110036, China; College of Medicine and Biological Information Engineering, Northeastern University, Shenyang, Liaoning, 110000, China; College of Medicine and Biological Information Engineering, Northeastern University, Shenyang, Liaoning, 110000, China; College of Artificial Intelligence, Dalian Maritime University, Dalian, Liaoning, 116026, China; School of Software, Dalian University of Technology, Dalian, Liaoning, 116024, China

## Abstract

**Motivation:**

N6-methyladenine (6 mA) is an important epigenetic modification of DNA that regulates biological processes such as gene expression, transcription, replication, DNA repair, and cell cycle without altering the DNA sequence. It also plays a key role in many diseases including cancer and autoimmune diseases. Although experimental approaches such as SMRT sequencing and methylated DNA immunoprecipitation can identify 6 mA sites, they suffer from drawbacks including suboptimal sequencing quality, low signal-to-noise ratios, high costs, and time-consuming procedures. In recent years, deep learning approaches have demonstrated significant advantages in predicting 6 mA sites; however, their generalization ability still requires further improvement.

**Results:**

Inspired by the state space model Mamba, we propose a novel model for 6 mA site prediction, named Mamba6mA. In the Mamba6mA model, we design position-specific linear layers to replace traditional convolutional layers to facilitate capture specific positional information. Meanwhile, we construct a multi-scale feature extraction module and integrate features captured by sliding windows of different scales, feeding them into the classifier for prediction. Experimental results show that Mamba6mA achieves the best MCC on 9 out of 11 species datasets, surpassing existing state-of-the-art models. Ablation studies confirm that the position-specific linear layers and the multi-scale fusion module contribute MCC performance gains of 2.36% and 2.31%, respectively. Feature visualization analysis further reveals that the model effectively captures sequence patterns upstream and downstream of 6 mA sites providing a new technical approach for studying epigenetic modification mechanisms.

**Availability and implementation:**

The source code for Mamba6mA is available at: https://github.com/XploreAI-Lab/Mamba6mA.

## 1 Introduction

N6-methyladenine (6 mA) (Tang *et al.* 2018) is a crucial DNA epigenetic modification (Sadida *et al.* 2024) that can regulate genetic activity without altering the DNA sequence. Recent studies have shown that 6 mA is involved in fundamental biological processes such as gene expression, transcription, replication, DNA repair, and the cell cycle (Pukkila *et al.* 1983, Campbell and Kleckner 1990, Robbins-Manke *et al.* 2005), playing an important role in proliferation, development, and regulation. In addition, 6 mA modification is closely associated with various diseases, including cancer and autoimmune diseases (Xiao *et al.* 2018, Loyfer *et al.* 2023). Therefore, accurate identification of 6 mA sites is of great significance for gaining deeper insight into epigenetic modification mechanisms and uncovering disease-related epigenetic regulation.

A variety of experimental techniques have been applied to detect DNA methylation sites, including methylated DNA immunoprecipitation (MeDIP) (Mohn *et al.* 2009), liquid chromatography–tandem mass spectrometry (LC-MS/MS) (Song *et al.* 2005), and single-molecule real-time (SMRT) sequencing technology (Flusberg *et al.* 2010). While MeDIP and LC-MS/MS are widely used, their low resolution limits the precise localization of methylation sites. In contrast, SMRT sequencing enables genome-wide detection of 6 mA at single-nucleotide resolution, but it suffers from a low signal-to-noise ratio and imposes high costs and time requirements (Liu *et al.* 2019). To overcome these limitations of experimental approaches, computational prediction methods have been developed and applied (Ao *et al.* 2022).

Early prediction of DNA methylation sites primarily relied on traditional artificial intelligence models. Chen *et al.* (2019) utilized support vector machines to analyze the chemical properties of nucleotides, constructing a 6 mA site prediction model for the rice genome and developing the i6mA-Pred web server. Building on this direction, Kong and Zhang (2019) designed a bagging classifier, i6mA-DNCP, which leveraged artificially constructed DNA sequence features to further improve 6 mA site identification in the rice genome. Extending beyond a single specie, Lv *et al.* (2020) proposed a random forest model named iDNA-MS, which demonstrated strong performance on a DNA methylation dataset spanning 11 species.

In recent years, deep learning has advanced rapidly, contributing significantly to various domains such as image recognition and natural language processing (Bayoudh 2024), and has also shown strong potential in predicting DNA methylation sites, with various models demonstrating distinct advantages across species-specific datasets (Hamamoto *et al.* 2019). In 2019, Yu and Dai (2019) first proposed SNNRice6mA, which utilized a convolutional neural network (CNN) architecture specifically for predicting 6 mA sites in the rice genome. Subsequently, Li *et al.* (2021) designed the Deep6mA model by integrating CNNs and long short-term memory (LSTM) (Yu *et al.* 2019), enabling the extraction of both local sequence patterns and long-range contextual dependencies, and significantly improving prediction performance. The DeepTorrent model (Liu *et al.* 2021) incorporated the attention mechanism and transfer learning strategy, which further improved the performance of 6 mA site prediction. In parallel, BERT6mA (Tsukiyama *et al.* 2022), based on the Transformer architecture, achieved even better predictive performance and demonstrated the feasibility of applying natural language processing techniques in this field. The recently proposed CNN6mA model (Tsukiyama *et al.* 2023) introduced 1D convolutional layers with a cross-interaction network to jointly capture local sequence features and interdependencies, thereby improving both prediction accuracy and robustness. Additionally, Jin *et al.* (Yu *et al.* 2021) developed a multi-type DNA methylation prediction model, iDNA-ABT, which supports identification of 6 mA, 4mC, and 5hmC sites across multiple species by leveraging adaptive feature learning to enhance the recognition accuracy of diverse methylation types.

Although existing deep learning models have achieved excellent performance in 6 mA site prediction, there remains significant room for improvement in inference performance. In 2023, Gu and Dao (2023) introduced Mamba, a novel state space model (SSM) that overcomes the limited expressiveness of conventional SSM in sequence modeling. The core of the Mamba architecture lies in its input-dependent selective mechanism, which achieves precise information filtering by suppressing redundant segments while enhancing critical functional sites—a characteristic that aligns profoundly with the intrinsic logic of biological sequences. This architecture has shown significant advantages in bioinformatics, as evidenced by recent successful applications including BarcodeMamb (Gao and Taylor 2024) for species identification, ChiMamba (Zhang *et al.* 2024) for chromatin interaction prediction, and MambaCpG (Zhao *et al.* 2025) for imputing single-cell DNA methylation states. These works collectively highlight the outstanding value and growing potential of the Mamba framework in biological sequence analysis.

Based on Mamba, we developed Mamba6mA, a model for 6 mA site prediction that incorporates a multi-scale feature extraction module and a feature fusion module. Specifically, in the feature extraction module, we replace the original convolutional layers in Mamba with position-specific linear layers to capture positional dependencies, and use linear layers with different window sizes to extract multi-scale features. The subsequent feature fusion module integrates these representations into a unified feature matrix. Experimental results on datasets from 11 species demonstrate that Mamba6mA outperforms existing state-of-the-art models in terms of MCC on 9 species. Ablation studies further validate the effectiveness of both the position-specific linear layers and the multi-scale feature extraction strategy in improving predictive performance.

## 2 Materials and methods

### 2.1 Dataset

We adopted the 6 mA benchmark dataset proposed by Lv *et al.* (2020) in iDNA-MS for model training and evaluation. This dataset comprises DNA sequences from 11 species. Each sequence is 41 bp in length and is labeled as either a positive or negative sample depending on whether its center nucleotide is a 6 mA site. For each species, the numbers of positive and negative samples are balanced. First, CD-HIT clustering was performed at an 80% sequence identity threshold to remove redundant sequences, retaining only one representative sequence from each cluster to prevent data leakage. The dataset is randomly divided into training and testing sets in a 1:1 ratio. Detailed sample statistics are provided in Table 1.

**Table 1:** Dataset.

Species	Positive sample	Negative sample
*Arabidopsis thaliana*	31 873	31 873
*Caenorhabditis elegans*	7961	7961
*Casuarina equisetifolia*	6066	6066
*Drosophila melanogaster*	11 191	11 191
*Fragaria vesca*	3102	3102
*Homo sapiens*	18 335	18 335
*Rosa chinensis*	599	599
*Saccharomyces cerevisiae*	3786	3786
*Thermus thermophilus*	107 600	107 600
*Thermus SUP5-1*	3379	3379
*Xanthomonas oryzae pv. oryzicola BLS256*	17 215	17 215

### 2.2 Model architecture

**Figure 1:**
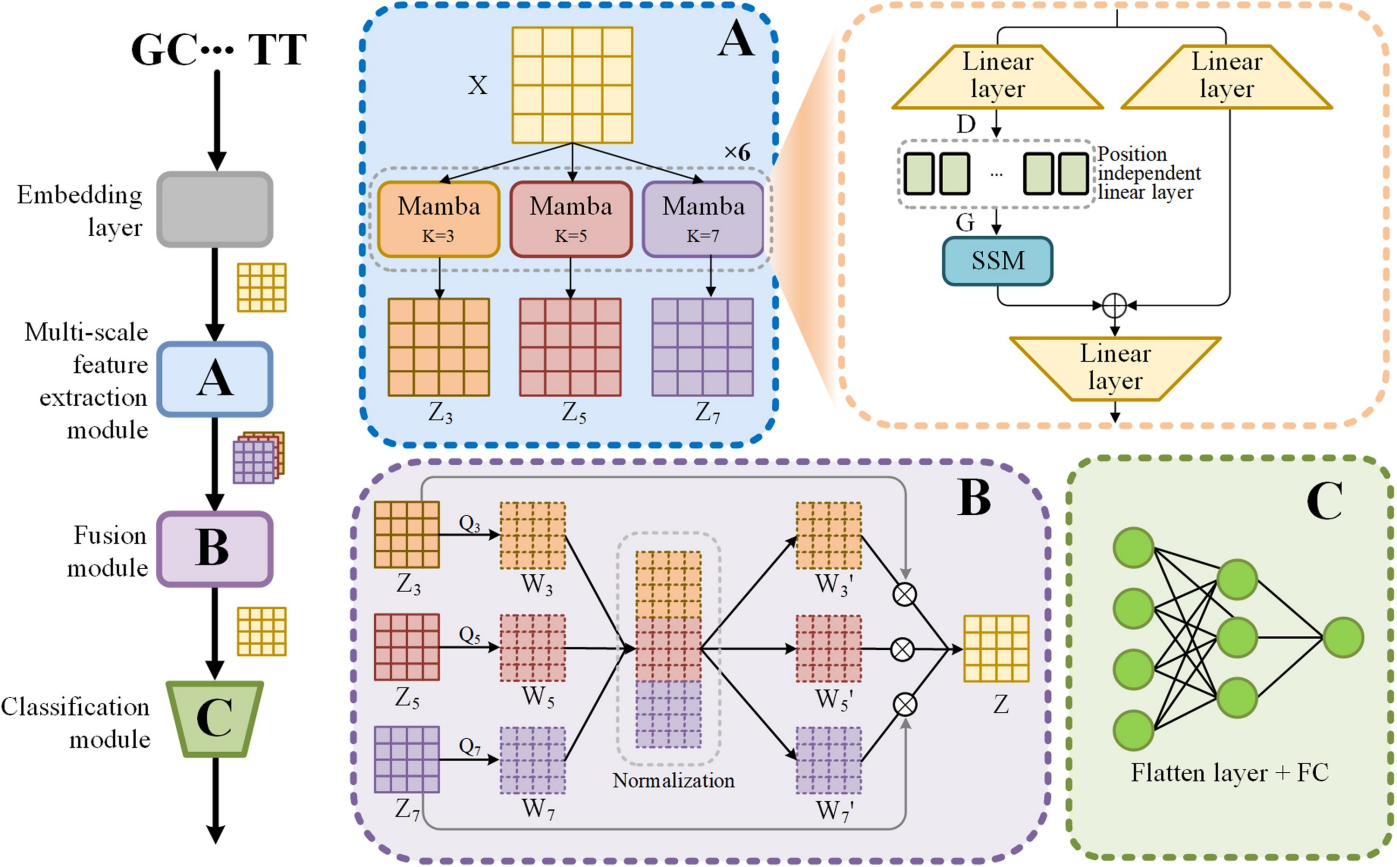
Overall architecture of Mamba6mA. (A) Multi-scale feature extraction module, which applies three parallel extraction branches with different window sizes to the input sequence, generating embeddings at multiple scales. (B) Feature fusion module, which fuses the multi-scale embeddings into a unified feature matrix. (C) Classification module, which predicts the output through a flatten layer followed by fully connected layers.

The overall architecture of proposed Mamba6mA model is shown in Fig. 1, which consists of three main components: the multi-scale feature extraction module (Fig. 1A), the feature fusion module (Fig. 1B), and the classification module (Fig. 1C). Prior to feature extraction, the input DNA sequence is passed through an embedding layer. Each nucleotide (A, C, G, T) in a DNA sequence of length *L* is first converted into a corresponding index and then mapped (Paszke *et al.* 2017) to an *N*-dimensional feature vector through a randomly initialized embedding layer that is jointly optimized with other network parameters during training, resulting in a matrix $X\in R^{L\times N}$. The multi-scale feature extraction module uses the Mamba architecture equipped with position-specific linear layers of varying window sizes to extract feature matrices at different scales, producing feature matrices $Z_{3}$, $Z_{5}$, and $Z_{7}$. These multi-scale features are subsequently aggregated by the fusion module into a unified feature matrix *Z*. Finally, the classification module predicts the label of the central nucleotide of the input sequence based on *Z*. A detailed description of each module’s structure and functionality is provided below.

#### 2.2.1 Multi-scale feature extraction module

The multi-scale feature extraction module consists of three groups of Mamba blocks at different scales. Each Mamba block comprises six Mamba layers stacked sequentially. In each Mamba layer, the embedded input $X\in R^{L\times N}$ is first projected via a linear layer into $D\in R^{L\times M}$, where *M* is the hidden dimension of Mamba layers. Next, *D* is passed through a position-specific linear layer, which can be regarded as a set of *L* convolutional filters with independent parameters, one for each position. Prior to convolution, zero padding is applied to the matrix, and then *F* filters of window size *K*, denoted as $f_{i,p}{\left(K\right)}_{k,j}(1\leq k\leq K,1\leq p\leq F)$, are applied at each position *i* in the sequence to produce the output matrix $G\in R^{L\times F}$. The value of $g_{i,p}$ is calculated as follows:


(1) $$g_{i,p}=\sum_{k=1}^{K}\sum_{j=1}^{M}f_{i,p}{\left(K\right)}_{k,j}\cdot d_{i-K/2+k-1,j}$$

In this study, the window sizes *K* for the three Mamba blocks are set to 3, 5, and 7, respectively. During training, the filters weights at specific positions are optimized, enabling the model to effectively capture position-specific information.

The matrix *G*, after activation by the SiLu function, is passed into the SSM module for further processing, as follows:


(2) $$h_{t+1}=\bar{A}\cdot h_{t}+\bar{B}\cdot G_{t}$$

(3) $$z_{t}=C\cdot h_{t}$$

Here, $G_{t}$ denotes the input to the SSM at time step *t*, *h* represents the state, and *z* denotes the output. Matrix $\bar{A}$ is the discretized state transition matrix, defining how the current state influences the next state. Matrix $\bar{B}$ determines how the input affects the system state, and matrix *C* maps the state to the output. All three matrices are learnable parameters. $\bar{A}$ and $\bar{B}$ are obtained by converting the continuous-time system into a discrete-time system using zero-order hold. Unlike traditional models based on state-space equations, Mamba dynamically computes matrices *B* and *C* during inference based on the input data.

Finally, after normalization, residual connection, and dimensionality reduction through a linear layer, the output feature matrix $Z_{k}\in R^{L\times N}$ is obtained.

#### 2.2.2 Feature fusion module

To fuse the feature matrices $Z_{3}$, $Z_{5}$, and $Z_{7}$ obtained from three parallel groups of Mamba layers, the fusion module (Fig. 1B) introduces three learnable *N*-dimensional vectors $Q_{3}$, $Q_{5}$, and $Q_{7}$. These vectors are broadcast along the sequence length dimension and then element-wise multiplied with the corresponding feature matrices to obtain the feature weight matrices, as follows:


(4) $$W_{3}=Q_{3}\cdot Z_{3},\;W_{5}=Q_{5}\cdot Z_{5},\;W_{7}=Q_{7}\cdot Z_{7}$$

The resulting weight matrices $W_{3}$, $W_{5}$, and $W_{7}$ are concatenated along the feature dimension and normalized using an exponential softmax operation. The normalized matrix is then split back into three parts, $W_{3}^{\prime }$, $W_{5}^{\prime }$, and $W_{7}^{\prime }$, which represent the normalized feature weight matrices for each scale:


(5) $$W_{3}^{\prime };W_{5}^{\prime };W_{7}^{\prime }=\text{Split}(\text{Softmax}(\text{Concat}(W_{3};W_{5};W_{7})))$$

The normalized weight matrices $W_{3}^{\prime }$, $W_{5}^{\prime }$, and $W_{7}^{\prime }$ are used to perform weighted multiplication with the original feature matrices $Z_{3}$, $Z_{5}$, and $Z_{7}$, respectively. The weighted feature matrices are then summed to obtain the final fused feature matrix *Z*:


(6) $$Z=W_{3}^{\prime }\cdot Z_{3}+W_{5}^{\prime }\cdot Z_{5}+W_{7}^{\prime }\cdot Z_{7}$$

The feature fusion module integrates features extracted at different scales in a weighted manner, effectively incorporating multi-scale features while preserving position-specific information across sequence positions, thereby enhancing the model’s expressive capacity and improving prediction accuracy.

#### 2.2.3 Classification module

In the classification module, the fused feature matrix *Z* is first flattened using a Flatten layer and then passed through a binary classification head for methylated and non-methylated sites, composed of three fully connected layers with dimensions 384, 16, and 1, respectively. The first two fully connected layers use ReLU as activation function, and apply dropout with a rate of 0.2. Finally, the output of the third fully connected layer is transformed using a Sigmoid activation function to generate the final prediction.

### 2.3 Training and evaluation

In this study, we used 5-fold cross-validation for model training and validation, with a batch size of 64. The model was optimized using the Adam optimizer (Kingma 2014). For species with larger sample sizes (*Arabidopsis thaliana* and *T. thermophile*), the learning rate was set to $1\times 10^{-5}$, while for the remaining species with smaller sample sizes, it was set to $5\times 10^{-5}$. Training was terminated if the validation accuracy did not improve for 30 consecutive epochs. All experiments were conducted on a system running Ubuntu 20.04, using Python 3.10.11, PyTorch (pytorch-cuda11.8), an NVIDIA RTX 3090 GPU, and 128GB of memory.

We adopted commonly used metrics to evaluate the performance of our model and other existing methods: Accuracy (ACC), Matthews Correlation Coefficient (MCC), Sensitivity (SN), and Specificity (SP). The calculation formulas are provided in Table 2, where TP, FN, TN, and FP denote the numbers of true positive, false negative, true negative, and false positive samples, respectively. Both ACC and MCC reflect the overall performance of the model. SN indicates the proportion of true methylated samples correctly predicted by the model, while SP measures the proportion of correctly identified non-methylated samples.

**Table 2:** Evaluation metrics.

Index	Equation
ACC	$\frac{\text{TP}+\text{TN}}{\text{TP}+\text{TN}+\text{FP}+\text{FN}}$
MCC	$\frac{\text{TP}\times \text{TN}-\text{FP}\times \text{FN}}{\sqrt{\left(\text{TP}+\text{FP})(\text{TP}+\text{FN})(\text{TN}+\text{FP})(\text{TN}+\text{FN}\right)}}$
SN	$\frac{\text{TP}}{\text{TP}+\text{FN}}$
SP	$\frac{\text{TN}}{\text{TN}+\text{FP}}$

## 3 Results

### 3.1 Comparison with existing methods

**Figure 2:**
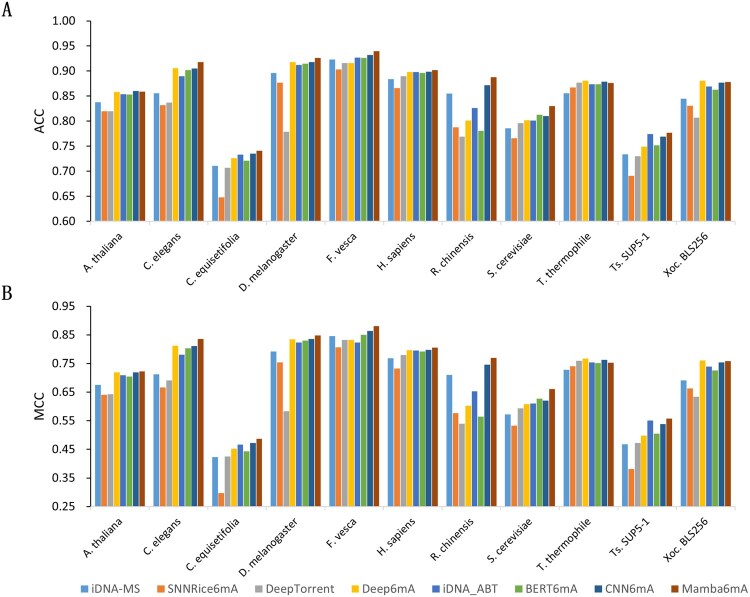
Performance comparison between Mamba6mA and other existing methods. (A) and (B) present the ACC and MCC values of Mamba6mA and other baseline methods on 11 independent benchmark datasets, respectively.

To ensure a fair comparison, we evaluated Mamba6mA against seven existing models and handled the baseline data in two distinct ways. Specifically, iDNA-ABT (Lv *et al.* 2020) and CNN6mA (Tsukiyama *et al.* 2023) were reproduced using official code under a uniform experimental framework. In contrast, the performance data for iDNA-MS (Lv *et al.* 2020), SNNRice6mA (Lv *et al.* 2020), DeepTorrent (Lv *et al.* 2020), Deep6mA (Lv *et al.* 2020), and BERT6mA (Lv *et al.* 2020) were taken directly from their original publications or authoritative reproduction studies. This methodology guarantees that all comparative data are consistent and equitably treated. Among these seven models, iDNA-MS is based on a random forest algorithm, while the remaining six are deep learning models. The comparison results are shown in Fig. 2 and Table 1, available as supplementary data at *Bioinformatics* online. On the datasets from 11 species, Mamba6mA achieved the highest ACC in 8 species (Fig. 2A), and ranked second-best on *A. thaliana* and Xoc.BLS256. For *T. thermophile*, it is slightly behind Deep6mA, CNN6mA, and DeepTorrent. With respect to the MCC (Fig. 2B), Mamba6mA achieved the best performance on 9 species, and performed comparably to the top models on the remaining two datasets (*T. thermophile* and Xoc. BLS256). These results demonstrate the strong learning capability and good robustness of Mamba6mA.

### 3.2 Ablation study

In the task of methylation site prediction, the positional information of nucleotides is a determinant of prediction accuracy (Saglam and Akgül 2024). The original Mamba architecture (Gu and Dao 2023), which uses 1D convolutional layers with shared parameters, is limited in its ability to capture position-specific information. To address this limitation, we replaced the 1D convolutional layers with position-specific linear layers. To assess the impact of this architectural modification, we conducted comparative experiments between the original Mamba architecture and the modified version incorporating position-specific linear layers across datasets from 11 species.

**Figure 3:**
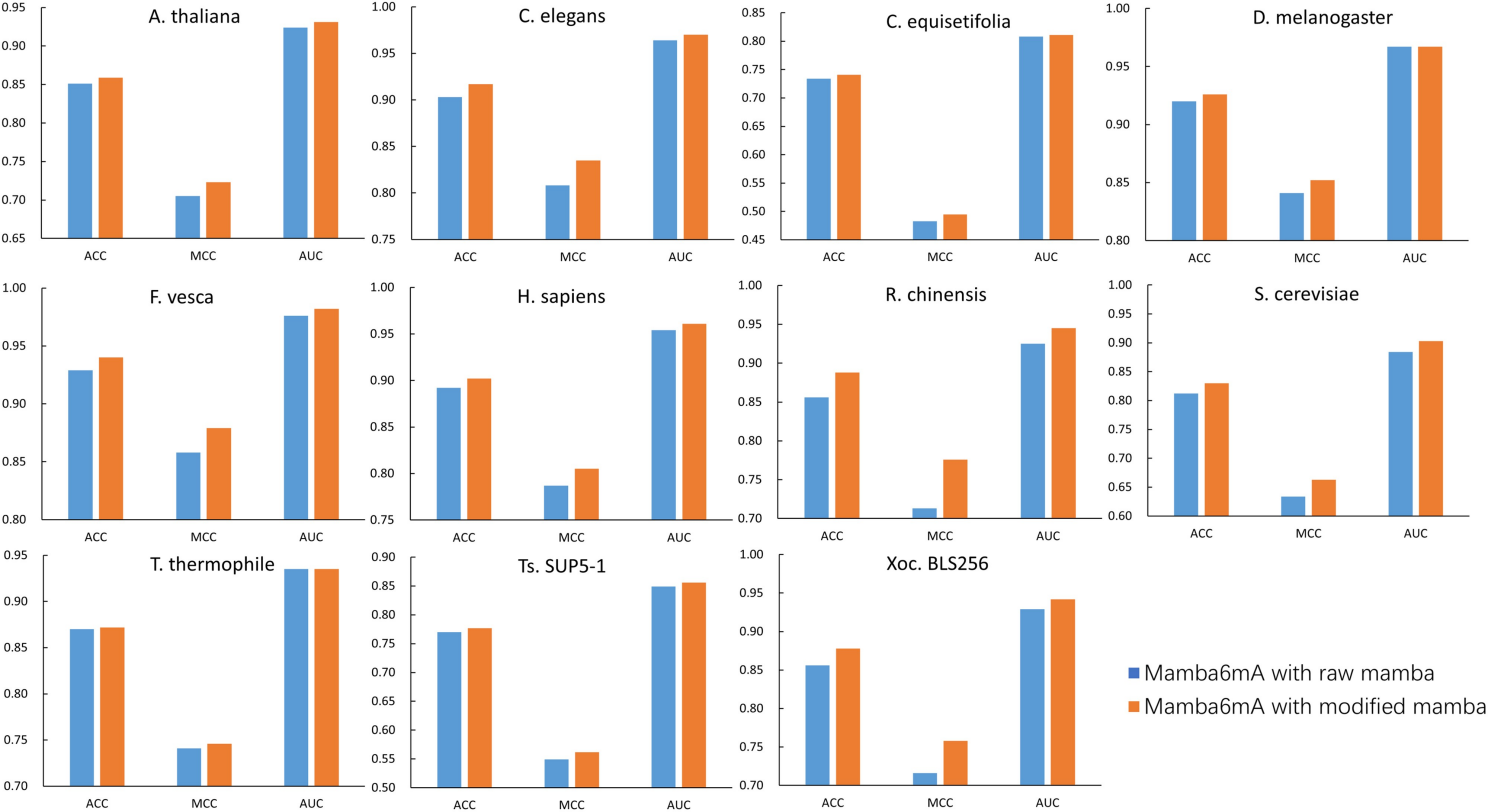
Comparison of ACC, MCC, and AUC between the traditional Mamba architecture based on standard convolutional layers and the modified Mamba architecture incorporating position-specific linear layers across 11 species.

As shown in Fig. 3 and Table 8, available as supplementary data at *Bioinformatics* online, Mamba6mA consistently outperformed the original Mamba model across all 11 species, achieving average improvements of 1.25% in ACC and 2.36% in MCC. For the AUC metric, Mamba6mA demonstrated advantages in 9 out of 11 species, with an average gain of 1.45%. These results confirm that the proposed position-specific mechanism substantially enhances the model’s ability to capture localized patterns in DNA sequences, thereby enabling more accurate extraction of methylation-related features.

**Figure 4:**
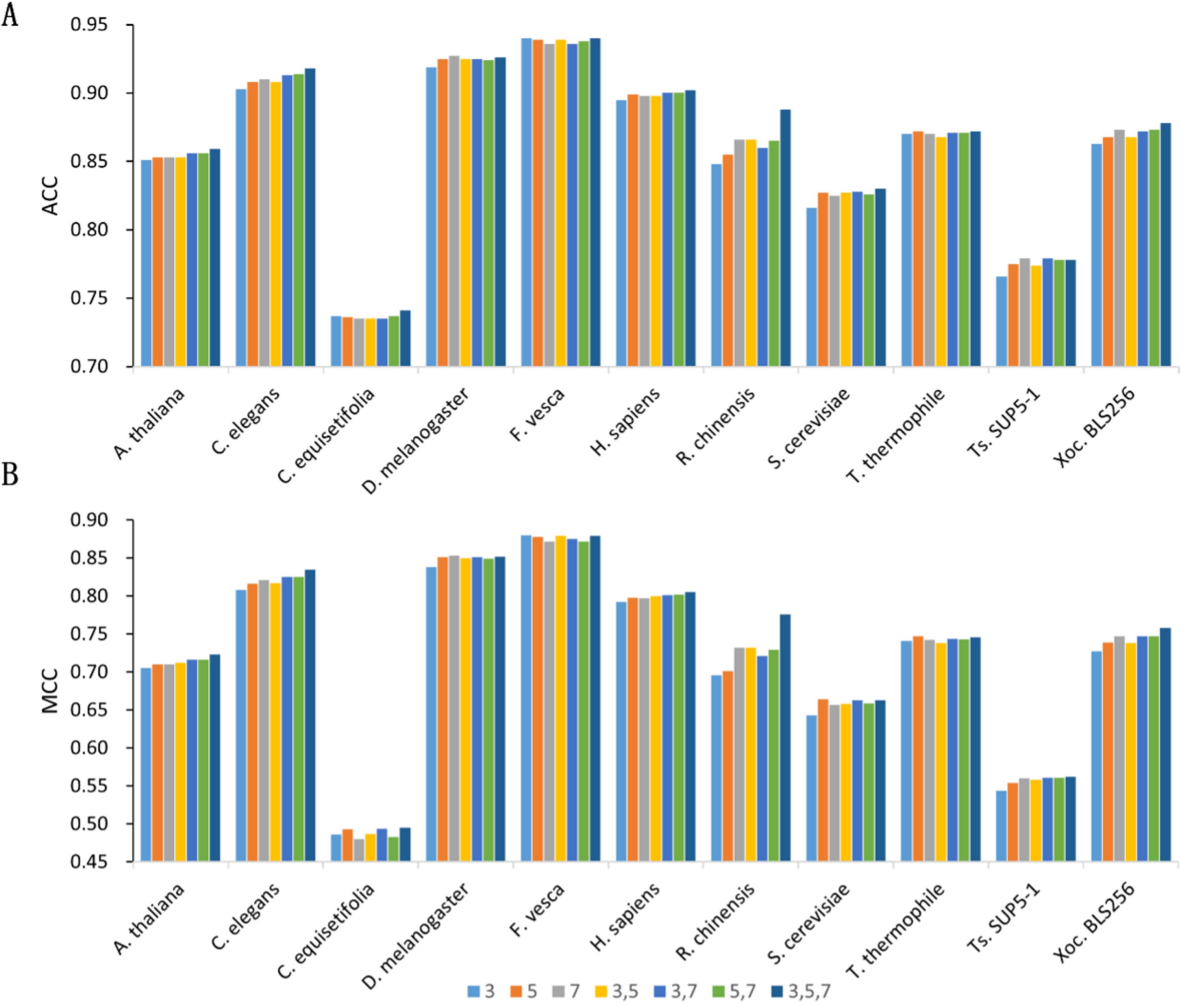
Performance comparison between single-scale and multi-scale combinations. (A) and (B) show the ACC and MCC comparisons of single scales (K=3, K=5, and K=7) and their multi-scale combination across 11 independent benchmark datasets.

To capture nucleotide patterns at different scales, we used linear layers with multiple window sizes in the Mamba module for feature extraction. We investigated the optimal combination of multi-scale linear layers for extracting position-specific features. Specifically, we constructed Mamba modules with position-specific linear layers using window sizes of 3, 5, and 7, respectively, and tested the performance of Mamba6mA on the test sets using these individual modules or their combinations. As shown in Fig. 4, Tables 9 and 10, available as supplementary data at *Bioinformatics* online, when using a single-window Mamba module, the ACC, MCC, and AUC values of Mamba6mA consistently increased with larger window sizes, possibly due to the richer contextual information captured by longer windows. In the case of Mamba modules with multiple window sizes, the combination of window sizes 3, 5, and 7 achieved the highest average ACC, MCC, and AUC, indicating that multi-scale feature extraction effectively enhances the model’s predictive performance.

### 3.3 Visualization of discriminative ability between positive and negative samples

**Figure 5:**
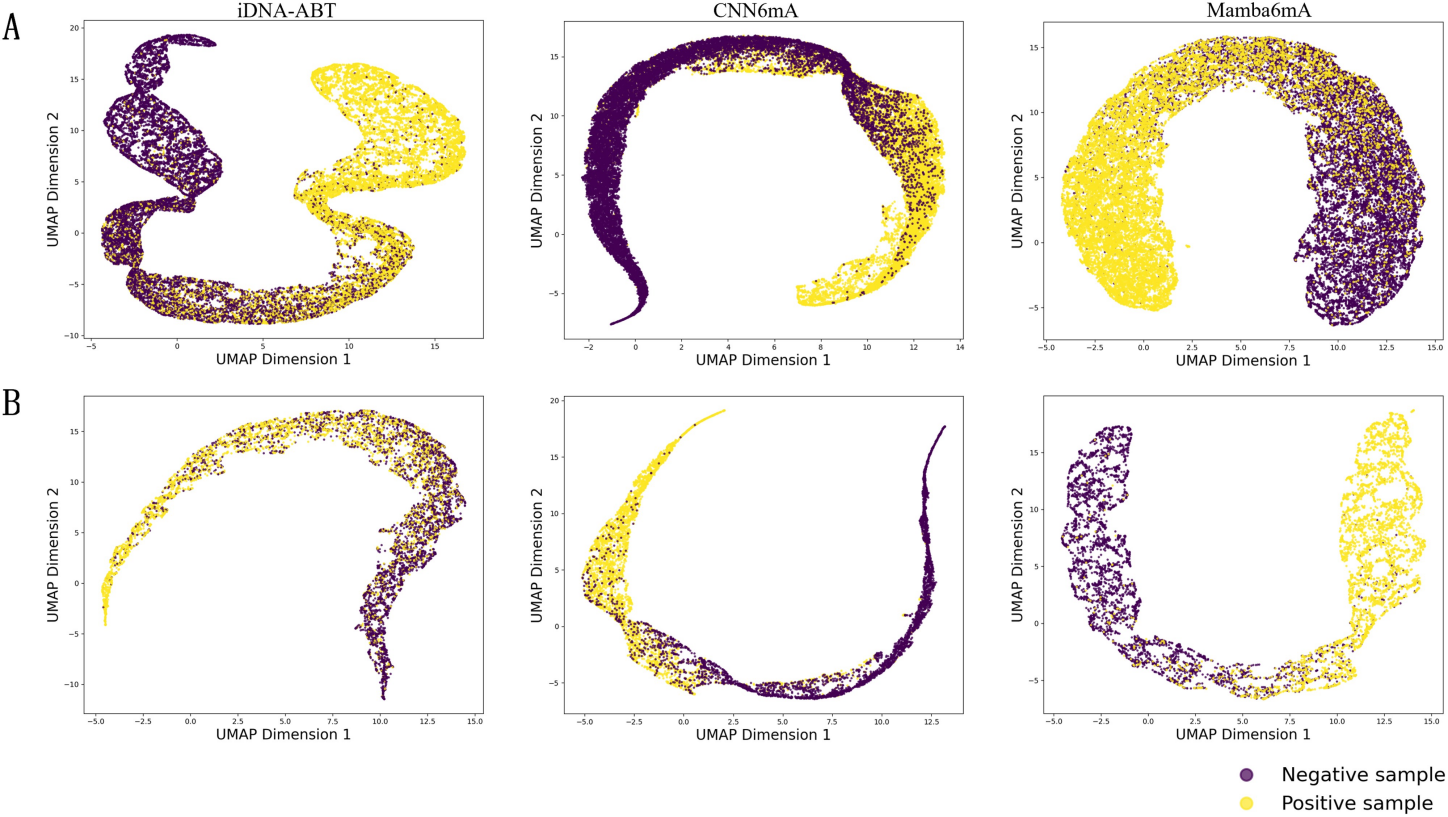
UMAP visualizations of iDNA-ABT, CNN6mA, and Mamba6mA. (A) Visualization results on the *A. thaliana* dataset. (B) Visualization results on the *C. elegans* dataset.

To evaluate the model’s ability to learn discriminative features, we extracted the outputs from the penultimate feedforward layer across the test sets of all 11 species and projected them into a 2D space using the UMAP dimensionality reduction technique (McInnes *et al.* 2018), enabling visualization and interpretation of the latent feature distribution. For comparison, UMAP visualizations were also performed on iDNA-ABT and CNN6mA using their publicly available code and hyperparameters under identical experimental settings. As shown in Fig. 5A and B, the spatial distributions of positive and negative samples for the three models on the *A. thaliana* and *Caenorhabditis elegans* datasets are presented (UMAP visualizations for other datasets are provided in Figs 1 and 2, available as supplementary data at *Bioinformatics* online). While all three models exhibit a degree of class separation, iDNA-ABT and CNN6mA show noticeable overlap near the decision boundary, whereas Mamba6mA achieves a clearer separation. This more distinct boundary in feature space indicates that our model learns more discriminative representations from the training samples.

### 3.4 Interpretation of the prediction mechanism based on positional relationships

**Figure 6:**
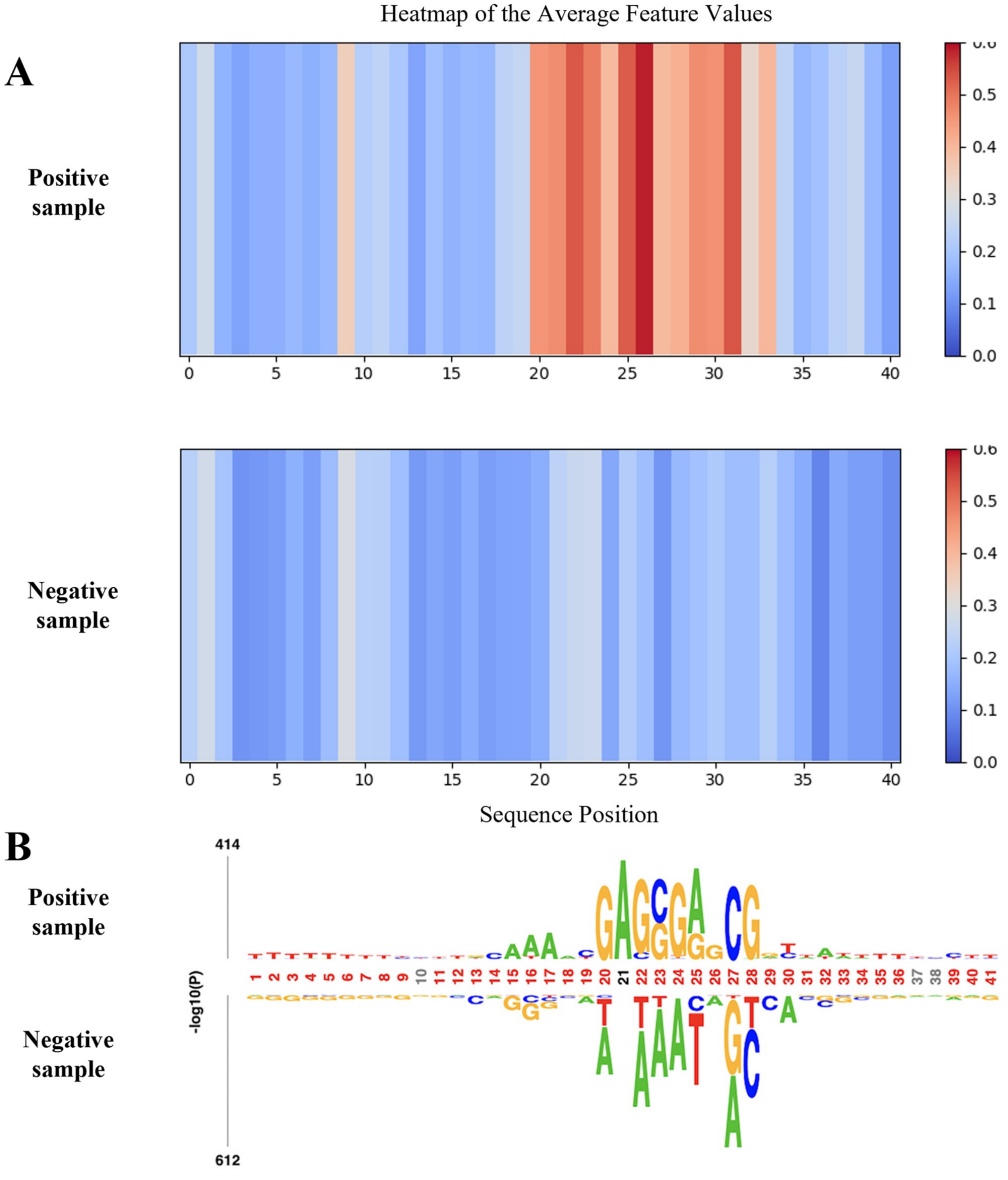
(A)Visualization of position-wise feature intensities for positive and negative samples in *H. sapiens*. (B) Nucleotide preference analysis of *H. sapiens* sequences using the pLogo, illustrating enrichment patterns for positive and negative samples.

To investigate whether the model captures sequence motif information during 6 mA identifying, we visualized the representations of positive and negative samples from the *Homo sapiens* dataset. Specifically, after feeding the samples into the model, we extracted the feature matrix $Z\in R^{L\times N}$ from the layer preceding the classification module, where *L* denotes the sequence length and *N* is the feature dimension. By averaging across the feature dimension, we obtained a position-wise feature intensity vector for each sample. We then computed the mean feature values $\bar{Z}$ separately for positive and negative samples based on their ground-truth labels and visualized these values using a heatmap. As shown in Fig. 6A, the horizontal axis represents positions along the input sequence, and the color indicates the magnitude of the feature intensities. The heatmap reveals that positive samples exhibit strong localized signals, with notably higher intensities between one nucleotide upstream of the methylation site (position 20) and ten nucleotides downstream (position 31). This suggests that the model attends primarily to this region, which may contain sequence motifs indicative of 6 mA sites. This observation is biologically plausible, as previous studies (Xiao *et al.* 2018) have reported that guanine (G) occurs with a frequency above 50% at one base upstream or downstream of 6 mA modification sites, forming a core sequence motif (GAG) flanking 6 mA sites, which aligns well with the feature differences observed at positions 20–22 in our heatmap. In contrast, negative samples show a more uniform and weaker distribution of feature intensities across the nucleotides, indicating the absence of informative signals in this region.

To further validate the key sequence features identified by the model, we used the pLogo tool (O’shea *et al.* 2013) to visualize the positive and negative samples in the *H. sapiens* dataset, as shown in Fig. 6B. pLogo is a widely used tool for detecting nucleotide sequence motifs and can reveal the distribution characteristics of nucleotide frequencies near 6 mA sites, capable of highlighting positional enrichment patterns of 6 mA by computing information content; a higher frequency of a specific nucleotide at a given position corresponds to lower entropy and greater motif significance. For positive samples, we observed elevated scores between positions 20 and 28, suggesting the presence of conserved sequence motifs in this region. This finding aligns well with the highly activated region revealed by the heatmap in Fig. 6A. Therefore, we infer that Mamba6mA identifies 6 mA sites by assessing the presence and strength of sequence motifs within key positional regions, thereby distinguishing positive from negative samples. These results demonstrate that Mamba6mA effectively captures sequence features associated with methylation, providing reliable information for the prediction of DNA methylation states.

## 4 Conclusion

The Mamba6mA model proposed in this study significantly enhances the accuracy of DNA 6 mA site prediction by incorporating position-specific linear layers and multi-scale feature extraction modules. This multi-scale architecture is biologically and methodologically motivated: 3 bp corresponds to codon length and minimal motif scale, while 5 bp and 7 bp cover common DNA motif ranges. The multi-scale design enables capturing diverse sequence features, and empirical tests confirmed that larger windows yield diminishing returns with significantly increased computational cost. Evaluated on datasets from 11 species, Mamba6mA outperformed existing state-of-the-art models in terms of MCC on 9 species. Ablation studies further confirmed the effectiveness of its architectural components. Sample visualizations and motif analyses revealed that Mamba6mA effectively captures sequence features associated with methylation. It is worth noting that we attempted to compare with pre-trained DNA language models such as DNABERT. However, these models failed to converge effectively in our 6 mA prediction task, likely due to the unique feature patterns of 6 mA methylation and the limited fine-tuning data available. Additionally, it should be noted that the negative samples used for model training were defined as sites where no 6 mA modification was detected by SMRT sequencing technology, which may include potential false negatives due to technical limitations. This label noise could affect both model performance and the reliability of evaluation metrics. Future research may focus on further optimizing the model architecture, improving the quality of training labels through more stringent experimental validation, and extending its application to the prediction of other types of epigenetic modifications.

## Supplementary Material

btag060_Supplementary_Data

## Data Availability

The source code for Mamba6mA is available at: https://github.com/XploreAI-Lab/Mamba6mA.
