# Supplementary material for: Mamba6mA: a Mamba-based DNA N6-methyladenine site prediction model
Source: Bioinformatics. 2026 Feb 5;42(3):btag060. doi: 10.1093/bioinformatics/btag060 (PMC12960908; doi:10.1093/bioinformatics/btag060)
Supplement: btag060_Supplementary_Data [file btag060_supplementary_data.pdf]

Supplemental Material

Table S1. The comparison of Mamba with other existing models in terms of ACC on 11 datasets.

**Dataset**	**iDNA-MS**	**SNNRice6mA**	**DeepTorrent**	**Deep6mA**	**iDNA_ABT**	**BERT6mA**	**CNN6mA**	**Mamba6mA**
A. thaliana	0.838	0.820	0.820	0.858	0.854	0.853	**0.860**	<u>0.859</u>
C. elegans	0.856	0.832	0.837	<u>0.906</u>	0.890	0.902	0.905	**0.918**
C. equisetifolia	0.711	0.648	0.707	0.726	0.733	0.721	<u>0.735</u>	**0.742**
D. melanogaster	0.896	0.877	0.779	<u>0.918</u>	0.912	0.915	<u>0.918</u>	**0.924**
F. vesca	0.923	0.903	0.916	0.916	0.927	0.926	<u>0.932</u>	**0.940**
H. sapiens	0.884	0.866	0.890	0.898	0.898	0.896	<u>0.899</u>	**0.902**
R. chinensis	0.855	0.788	0.769	0.801	0.826	0.781	<u>0.872</u>	**0.888**
S. cerevisiae	0.786	0.766	0.796	0.802	0.801	<u>0.813</u>	0.810	**0.830**
T. thermophile	0.856	0.867	0.877	**0.881**	0.874	0.874	<u>0.879</u>	0.876
Ts. SUP5-1	0.734	0.691	0.730	0.749	<u>0.774</u>	0.752	0.769	**0.777**
Xoc. BLS256	0.845	0.831	0.807	**0.881**	0.869	0.863	0.877	<u>0.878</u>

[1] The best performance is highlighted in bold, and the second best is underlined.

Table S2. The comparison of Mamba with other existing models in terms of AUC on 11 datasets.

**Species**	**iDNA-MS**	**SNNRice6mA**	**DeepTorrent**	**Deep6mA**	**iDNA_ABT**	**BERT6mA**	**CNN6mA**	**Mamba6mA**
A. thaliana	0.911	0.899	0.899	<u>0.931</u>	0.918	0.927	**0.932**	0.930
C. elegans	0.935	0.913	0.913	0.962	0.943	0.962	<u>0.966</u>	**0.967**
C. equisetifolia	0.779	0.712	0.781	0.798	0.790	0.799	<u>0.807</u>	**0.819**
D. melanogaster	0.956	0.942	0.875	0.968	0.954	0.967	<u>0.968</u>	**0.971**
F. vesca	0.977	0.967	0.966	0.965	0.954	0.976	<u>0.978</u>	**0.978**
H. sapiens	0.950	0.941	0.957	**0.963**	0.951	0.962	<u>0.962</u>	0.961
R. chinensis	0.924	0.900	0.820	0.882	0.879	0.865	<u>0.937</u>	**0.948**
S. cerevisiae	0.868	0.846	0.873	0.886	0.871	0.890	<u>0.893</u>	**0.901**
T. thermophile	0.922	0.930	0.940	**0.944**	0.931	0.938	<u>0.942</u>	0.938
Ts. SUP5-1	0.813	0.755	0.801	0.828	0.836	0.834	<u>0.844</u>	**0.851**
Xoc. BLS256	0.921	0.914	0.915	**0.949**	0.926	0.936	<u>0.947</u>	0.940

[1] The best performance is highlighted in bold, and the second best is underlined.

Table S3. The comparison of Mamba with other existing models in terms of MCC on 11 datasets.

Species	iDNA-MS	SNNRice6mA	DeepTorrent	Deep6mA	iDNA_ABT	BERT6mA	CNN6mA	Mamba6mA
A. thaliana	0.676	0.641	0.643	0.719	0.709	0.705	0.719	**0.723**
C. elegans	0.712	0.666	0.691	0.812	0.781	0.803	0.811	**0.836**
C. equisetifolia	0.423	0.297	0.425	0.452	0.467	0.443	0.472	**0.487**
D. melanogaster	0.792	0.754	0.584	0.835	0.824	0.830	0.836	**0.848**
F. vesca	0.846	0.807	0.832	0.832	0.824	0.851	0.864	**0.881**
H. sapiens	0.769	0.733	0.780	0.797	0.796	0.792	0.798	**0.806**
R. chinensis	0.710	0.577	0.540	0.603	0.653	0.564	0.746	**0.770**
S. cerevisiae	0.572	0.533	0.594	0.608	0.610	0.627	0.620	**0.661**
T. thermophile	0.728	0.741	0.760	**0.768**	0.754	0.752	0.763	0.753
Ts. SUP5-1	0.468	0.382	0.472	0.498	0.551	0.505	0.539	**0.558**
Xoc. BLS256	0.691	0.663	0.634	**0.761**	0.739	0.726	0.754	0.759

[1] The best performance is highlighted in bold, and the second best is underlined.

Table S4. The comparison of Mamba with other existing models in terms of Precision on 11 datasets.

Species	iDNA-MS	SNNRice6mA	DeepTorrent	Deep6mA	iDNA_ABT	BERT6mA	CNN6mA	Mamba6mA
A. thaliana	0.847	0.877	0.837	0.857	0.886	0.857	0.870	**0.905**
C. elegans	0.848	0.897	0.807	**0.931**	0.900	0.896	0.890	0.926
C. equisetifolia	0.709	0.724	0.756	0.649	0.769	0.728	0.755	**0.775**
D. melanogaster	0.902	0.920	0.882	0.896	0.914	0.917	0.921	**0.943**
F. vesca	0.908	0.930	0.894	0.926	0.913	0.926	0.927	**0.937**
H. sapiens	0.901	0.901	0.870	0.907	0.890	0.900	0.901	**0.930**
R. chinensis	0.837	0.838	0.788	0.751	0.822	0.804	0.837	**0.898**
S. cerevisiae	0.805	0.857	0.769	0.817	0.841	0.821	0.819	**0.862**
T. thermophile	0.796	0.835	0.820	0.836	0.840	0.839	0.839	**0.855**
Ts. SUP5-1	0.731	**0.809**	0.701	0.794	0.747	0.742	0.781	0.808
Xoc. BLS256	0.859	0.855	0.819	0.739	**0.884**	0.874	0.874	0.851

[1] The best performance is highlighted in bold, and the second best is underlined.

Table S5. The comparison of Mamba with other existing models in terms of Recall/SN on 11 datasets.

Species	iDNA-MS	SNNRice6mA	DeepTorrent	Deep6mA	iDNA_ABT	BERT6mA	CNN6mA	Mamba6mA
A. thaliana	0.824	0.823	0.793	0.767	0.823	0.846	**0.846**	0.802
C. elegans	0.868	0.882	0.872	0.728	0.913	0.908	**0.924**	0.908
C. equisetifolia	**0.728**	0.718	0.689	0.645	0.590	0.707	0.696	0.685
D. melanogaster	0.890	0.904	0.871	0.630	**0.922**	0.915	0.913	0.912
F. vesca	0.930	0.923	0.914	0.903	0.920	0.925	0.938	**0.944**
H. sapiens	0.863	0.894	0.862	0.869	**0.908**	0.891	0.896	0.870
R. chinensis	**0.880**	0.809	0.727	0.806	0.769	0.743	0.787	0.860
S. cerevisiae	0.754	0.724	0.762	0.764	0.745	**0.801**	0.795	0.782
T. thermophile	**0.958**	0.933	0.940	0.941	0.941	0.925	0.938	0.906
Ts. SUP5-1	0.743	0.718	0.665	0.623	0.753	**0.772**	0.749	0.725
Xoc. BLS256	0.825	0.889	0.850	**0.941**	0.876	0.848	0.880	0.840

[1] The best performance is highlighted in bold, and the second best is underlined.

Table S6. The comparison of Mamba with other existing models in terms of SP on 11 datasets.

Species	iDNA-MS	iDNA_ABT	SNNRice6mA	DeepTorrent	Deep6mA	BERT6mA	CNN6mA	Mamba6mA
A. thaliana	0.851	0.894	0.846	0.872	0.894	0.859	0.873	**0.916**
C. elegans	0.844	0.899	0.792	**0.946**	0.898	0.895	0.886	0.927
C. equisetifolia	0.705	0.777	0.651	**0.823**	0.723	0.736	0.774	0.801
D. melanogaster	0.903	0.921	0.883	0.927	0.913	0.917	0.921	**0.945**
F. vesca	0.906	0.930	0.892	0.928	0.912	0.926	0.926	**0.937**
H. sapiens	0.905	0.902	0.871	0.911	0.888	0.901	0.902	**0.934**
R. chinensis	0.829	0.843	0.804	0.732	0.833	0.819	0.847	**0.908**
S. cerevisiae	0.817	**0.879**	0.771	0.829	0.859	0.825	0.824	0.875
T. thermophile	0.755	0.815	0.793	0.816	0.821	0.823	0.820	**0.846**
Ts. SUP5-1	0.726	0.830	0.716	**0.838**	0.745	0.732	0.790	0.828
Xoc. BLS256	0.865	0.849	0.812	0.667	0.885	0.878	0.873	**0.917**

[1] The best performance is highlighted in bold, and the second best is underlined.

Table S7. The comparison of Mamba with other existing models in terms of F1 Score on 11 datasets.

Species	iDNA-MS	SNNRice6mA	DeepTorrent	Deep6mA	iDNA_ABT	BERT6mA	CNN6mA	Mamba6mA
A. thaliana	0.835	0.849	0.815	0.810	0.853	0.852	**0.858**	0.851
C. elegans	0.858	0.890	0.839	0.817	0.906	0.902	0.907	**0.917**
C. equisetifolia	0.713	0.726	0.721	0.647	0.668	0.717	0.724	**0.727**
D. melanogaster	0.896	0.912	0.876	0.740	0.918	0.915	0.918	**0.927**
F. vesca	0.919	0.926	0.904	0.914	0.916	0.932	0.932	**0.941**
H. sapiens	0.882	0.898	0.866	0.888	0.899	0.896	0.899	**0.899**
R. chinensis	**0.858**	0.823	0.756	0.777	0.794	0.772	0.811	0.849
S. cerevisiae	0.779	0.785	0.765	0.790	0.790	0.811	0.807	**0.820**
T. thermophile	0.870	0.881	0.876	0.885	**0.888**	0.880	0.886	0.880
Ts. SUP5-1	0.737	0.761	0.682	0.698	0.750	0.757	0.765	**0.764**
Xoc. BLS256	0.842	0.872	0.834	0.828	0.880	0.861	0.877	**0.883**

[1] The best performance is highlighted in bold, and the second best is underlined.

Table S8. Comparison of Normal CNN and Parameter Independent CNN across ACC, MCC, and AUC

Dataset	ACC		MCC		AUC	
	Normal CNN	Parameter Independent CNN	Normal CNN	Parameter Independent CNN	Normal CNN	Parameter Independent CNN
A. thaliana	0.851	0.859	0.705	0.723	0.924	0.931
C. elegans	0.903	0.917	0.808	0.835	0.964	0.970
C. equisetifolia	0.734	0.741	0.483	0.495	0.808	0.811
D. melanogaster	0.920	0.926	0.841	0.852	0.967	0.967
F. vesca	0.929	0.940	0.858	0.879	0.976	0.982
H. sapiens	0.892	0.902	0.787	0.805	0.954	0.961
R. chinensis	0.856	0.888	0.713	0.776	0.925	0.945
S. cerevisiae	0.812	0.830	0.634	0.663	0.884	0.903
T. thermophile	0.870	0.872	0.741	0.746	0.935	0.935
Ts. SUP5-1	0.770	0.777	0.549	0.562	0.849	0.856
Xoc. BLS256	0.856	0.878	0.716	0.758	0.929	0.942

Table S9. The performance of Mamba model in ACC under single-scale and multi-scale combinations

**Dataset**	**3**	**5**	**7**	**3,5**	**3,7**	**5,7**	**3,5,7**
A. thaliana	0.851	0.853	0.853	0.853	0.856	0.856	**0.859**
C. elegans	0.903	0.908	0.910	0.908	0.913	0.914	**0.918**
C. equisetifolia	0.737	0.736	0.735	0.735	0.735	0.737	**0.741**
D. melanogaster	0.919	0.925	**0.927**	0.925	0.925	0.924	0.926
F. vesca	0.940	0.939	0.936	0.939	0.936	0.938	**0.940**
H. sapiens	0.895	0.899	0.898	0.898	0.900	0.900	**0.902**
R. chinensis	0.848	0.855	0.866	0.866	0.860	0.865	**0.888**
S. cerevisiae	0.816	0.827	0.825	0.827	0.828	0.826	**0.830**
T. thermophile	0.870	0.872	0.870	0.868	0.871	0.871	**0.872**
Ts. SUP5-1	0.766	0.775	0.779	0.774	**0.779**	0.778	0.778
Xoc. BLS256	0.863	0.868	0.873	0.868	0.872	0.873	**0.878**

[1] The best performance is highlighted in bold, and the second best is underlined.

Table S10. The performance of Mamba model in AUC under single-scale and multi-scale combinations

**Dataset**	**3**	**5**	**7**	**3,5**	**3,7**	**5,7**	**3,5,7**
A. thaliana	0.924	0.927	0.927	0.928	0.928	0.929	**0.931**
C. elegans	0.964	0.965	0.966	0.967	0.969	0.968	**0.970**
C. equisetifolia	0.801	0.806	0.808	0.806	0.806	0.810	**0.811**
D. melanogaster	0.965	0.967	0.967	0.968	0.968	**0.968**	0.967
F. vesca	0.982	0.982	**0.983**	0.982	0.982	0.982	0.982
H. sapiens	0.958	0.959	0.960	0.960	0.961	0.961	**0.961**
R. chinensis	0.925	0.931	0.935	0.936	0.935	0.935	**0.945**
S. cerevisiae	0.891	0.898	0.899	0.897	0.900	0.901	**0.903**
T. thermophile	0.935	0.934	0.933	0.933	0.934	0.931	**0.935**
Ts. SUP5-1	0.844	0.849	0.845	0.852	0.851	0.853	**0.856**
Xoc. BLS256	0.933	0.936	0.937	0.935	0.939	0.940	**0.942**

[1] The best performance is highlighted in bold, and the second best is underlined.

Table S11. The performance of Mamba model in MCC under single-scale and multi-scale combinations

**Dataset**	**3**	**5**	**7**	**3,5**	**3,7**	**5,7**	**3,5,7**
A. thaliana	0.705	0.710	0.710	0.712	0.716	<u>0.716</u>	**0.723**
C. elegans	0.808	0.816	0.821	0.817	0.825	<u>0.825</u>	**0.835**
C. equisetifolia	0.486	0.493	0.480	0.487	<u>0.494</u>	0.483	**0.495**
D. melanogaster	0.838	0.851	**0.853**	0.850	0.851	0.849	<u>0.852</u>
F. vesca	**0.880**	0.878	0.872	0.879	0.875	0.872	<u>0.879</u>
H. sapiens	0.792	0.798	0.797	0.800	0.801	<u>0.802</u>	**0.805**
R. chinensis	0.696	0.701	0.732	<u>0.732</u>	0.721	0.729	**0.776**
S. cerevisiae	0.643	**0.664**	0.657	0.658	0.663	0.659	<u>0.663</u>
T. thermophile	0.741	**0.747**	0.742	0.738	0.744	0.743	<u>0.746</u>
Ts. SUP5-1	0.544	0.554	0.560	0.558	0.561	<u>0.561</u>	**0.562**
Xoc. BLS256	0.727	0.739	0.747	0.738	0.747	<u>0.747</u>	**0.758**

[1] The best performance is highlighted in bold, and the second best is underlined.

Table S12. Ablation study on model configurations (A.thaliana).

Ablation Type	Configuration	ACC	MCC	AUC
Number of Layers	4 Layers	0.856	0.712	0.927
	6 Layers (ours)	0.859	0.723	0.930
	8 Layers	0.859	0.722	0.930
Hidden Dimension	64	0.876	0.696	0.928
	128	0.852	0.713	0.930
	256 (ours)	0.859	0.723	0.930
	512	0.859	0.721	0.929

Table S13. Ablation study on model configurations (H.sapient).

Ablation Type	Configuration	ACC	MCC	AUC
Number of Layers	4 Layers	0.896	0.795	0.955
	6 Layers (ours)	0.902	0.806	0.961
	8 Layers	0.902	0.803	0.962
Hidden Dimension	64	0.894	0.792	0.951
	128	0.893	0.792	0.957
	256 (ours)	0.902	0.806	0.961
	512	0.901	0.803	0.961

Table S14. Computational time (seconds) for training and inference with different context window configurations.

Dataset	Num of Mamba Block=1		Num of Mamba Block=2		Num of Mamba Block=3	
	Train	Inference	Train	Inference	Train	Inference
A. thaliana	84.60	19.30	162.26	32.46	245.22	48.26
C. elegans	21.16	4.65	42.50	9.39	64.15	13.70
C. equisetifolia	16.31	3.66	32.53	7.05	48.80	10.45
D. melanogaster	29.80	6.57	60.35	12.99	89.92	18.99
F. vesca	8.15	1.76	16.76	3.59	23.86	4.73
H. sapiens	48.42	11.11	97.98	21.12	147.35	31.26
R. chinensis	1.65	0.36	3.36	0.71	4.67	0.93
S. cerevisiae	10.36	2.24	20.19	4.28	29.18	5.72
T. thermophile	285.36	63.43	574.36	125.08	827.20	174.42
Ts. SUP5-1	9.57	2.22	18.09	3.94	27.19	5.68
Xoc. BLS256	45.44	10.03	88.03	17.45	138.52	29.48

Table S15. Efficiency comparison between Mamba6mA and Transformer.

Datasets	Training Time/Epoch (s)		Inference Time (s)		GPU Peak (MB)
	Mamba6mA	Transformer	Mamba6mA	Transformer	
A. thaliana	245.22	61.01	48.26	30.29	2021.7 / 1255.5
D. melanogaster	89.92	24.78	18.98	12.12	2021.7 / 1255.5
S. cerevisiae	29.18	8.12	5.72	3.87	2021.7 / 1255.5

Table S16. Performance comparison between Mamba6mA and its Transformer-replaced variant.

Datasets	Mamba6mA			with Transformer block		
	ACC	MCC	AUC	ACC	MCC	AUC
A. thaliana	0.859	0.723	0.930	0.851	0.704	0.915
C. elegans	0.918	0.836	0.967	0.905	0.809	0.961
C. equisetifolia	0.741	0.487	0.819	0.731	0.468	0.776
D. melanogaster	0.926	0.848	0.971	0.919	0.840	0.961
F. vesca	0.940	0.881	0.978	0.929	0.859	0.975
H. sapiens	0.902	0.806	0.961	0.891	0.785	0.953
R. chinensis	0.888	0.770	0.948	0.841	0.683	0.923
S. cerevisiae	0.830	0.661	0.901	0.817	0.641	0.879
T. thermophile	0.876	0.753	0.938	0.873	0.751	0.930
Ts. SUP5-1	0.777	0.558	0.851	0.765	0.537	0.838
Xoc. BLS256	0.878	0.759	0.940	0.871	0.743	0.932

Table S17. Performance comparison of different feature fusion strategies.

Species	ACC			MCC			AUC		
	**Mamba6mA**	**Attention**	**Average**	**Mamba6mA**	**Attention**	**Average**	**Mamba6mA**	**Attention**	**Average**
A. thaliana	0.859	0.854	0.850	0.723	0.709	0.702	0.930	0.924	0.920
C. elegans	0.918	0.909	0.911	0.836	0.818	0.823	0.967	0.967	0.967
C. equisetifolia	0.741	0.731	0.738	0.487	0.465	0.488	0.819	0.798	0.808
D. melanogaster	0.926	0.920	0.921	0.848	0.839	0.842	0.971	0.966	0.966
F. vesca	0.940	0.935	0.936	0.881	0.871	0.869	0.978	0.981	0.978
H. sapiens	0.902	0.896	0.897	0.806	0.794	0.796	0.961	0.959	0.959
R. chinensis	0.888	0.856	0.853	0.770	0.712	0.706	0.948	0.929	0.932
S. cerevisiae	0.830	0.818	0.827	0.661	0.638	0.658	0.901	0.890	0.899
T. thermophile	0.876	0.871	0.869	0.753	0.747	0.740	0.938	0.933	0.933
Ts. SUP5-1	0.777	0.773	0.773	0.558	0.550	0.551	0.851	0.852	0.851
Xoc. BLS256	0.878	0.874	0.872	0.759	0.748	0.745	0.940	0.941	0.938

Table S18. Performance comparison of different models using clustering metrics.

Species	iDNA-ABT			CNN6mA			Mamba6mA		
	**NMI**	**ARI**	**Purity**	**NMI**	**ARI**	**Purity**	**NMI**	**ARI**	**Purity**
A. thaliana	0.402	0.447	0.845	0.406	0.438	0.840	0.425	0.520	0.860
C. elegans	0.468	0.503	0.884	0.512	0.548	0.902	0.593	0.619	0.918
C. equisetifolia	0.147	0.173	0.690	0.150	0.250	0.709	0.188	0.232	0.741
D. melanogaster	0.553	0.559	0.841	0.523	0.618	0.843	0.615	0.722	0.925
F. vesca	0.588	0.622	0.741	0.603	0.629	0.928	0.663	0.764	0.937
H. sapiens	0.441	0.521	0.737	0.522	0.517	0.768	0.541	0.641	0.900
R. chinensis	0.407	0.389	0.722	0.326	0.412	0.739	0.460	0.561	0.875
S. cerevisiae	0.269	0.628	0.755	0.341	0.641	0.807	0.334	0.726	0.826
T. thermophile	0.467	0.526	0.847	0.552	0.537	0.881	0.495	0.541	0.867
Ts. SUP5-1	0.215	0.259	0.605	0.267	0.301	0.711	0.247	0.320	0.722
Xoc. BLS256	0.421	0.457	0.767	0.497	0.543	0.877	0.457	0.562	0.875

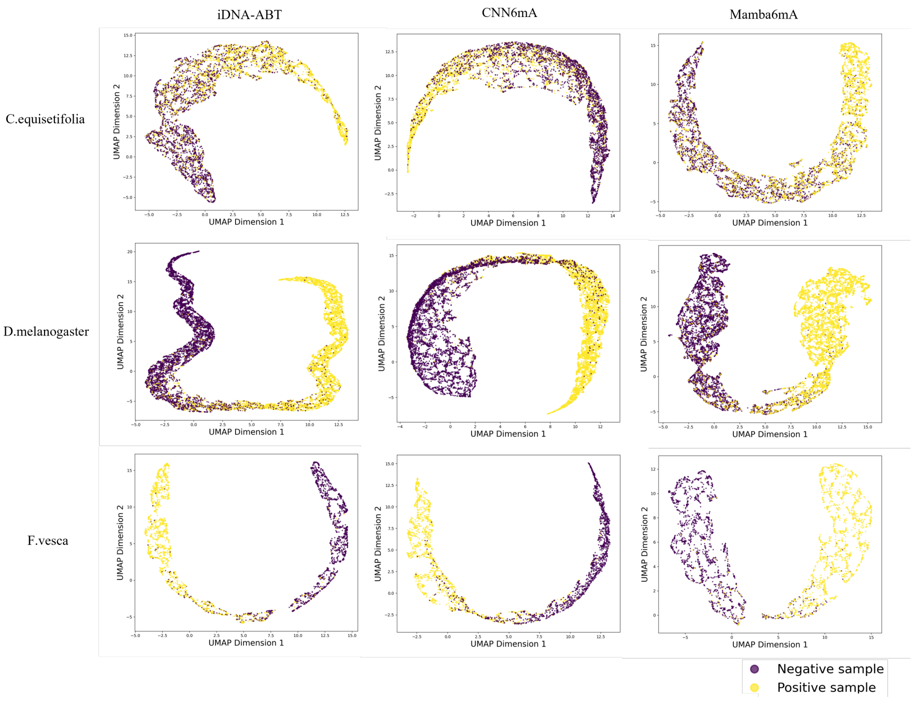

Fig. S1. Visualization of species datasets using Umap on

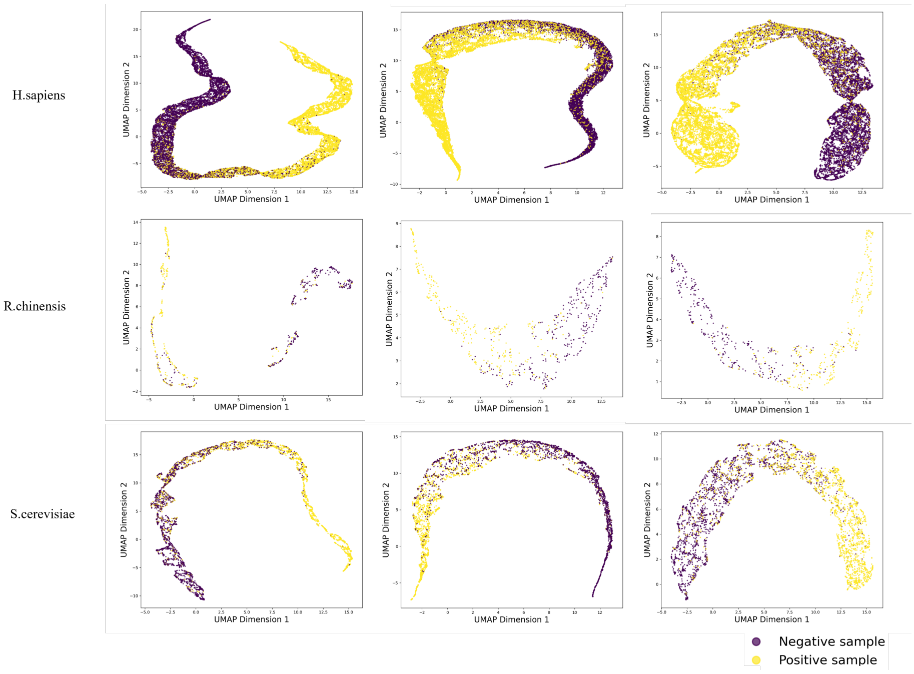

Fig. S2. Visualization of species datasets using Umap

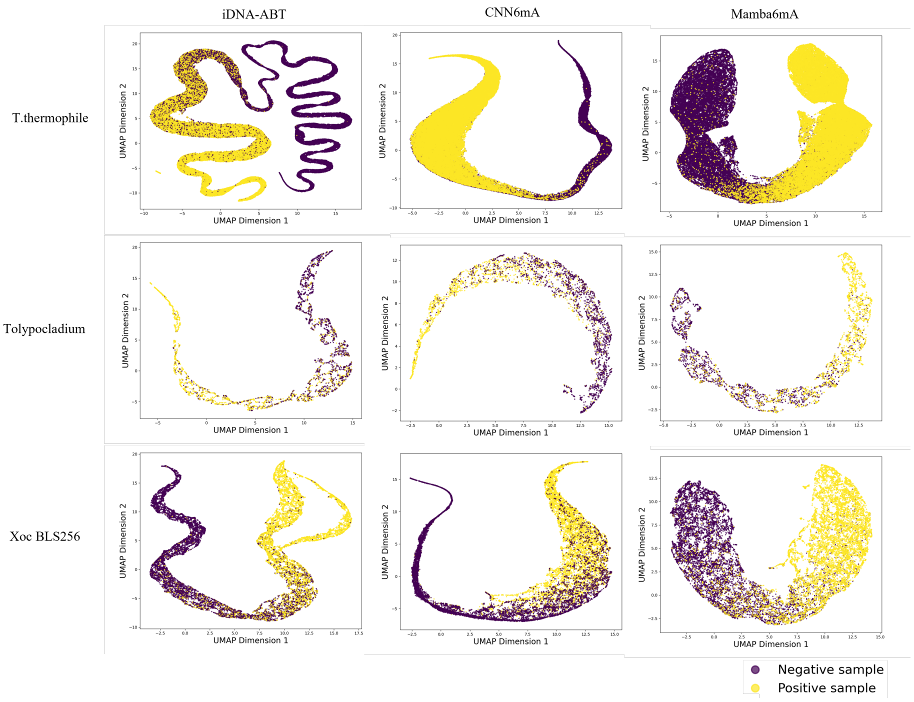

Fig. S3. Visualization of species datasets using Umap

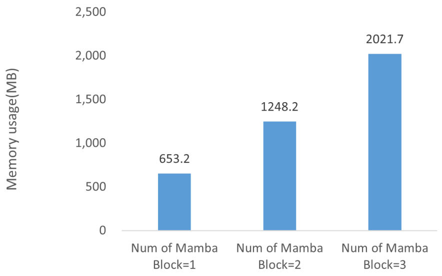

Fig. S4. The impact of the multi-scale Mamba module on memory usage.

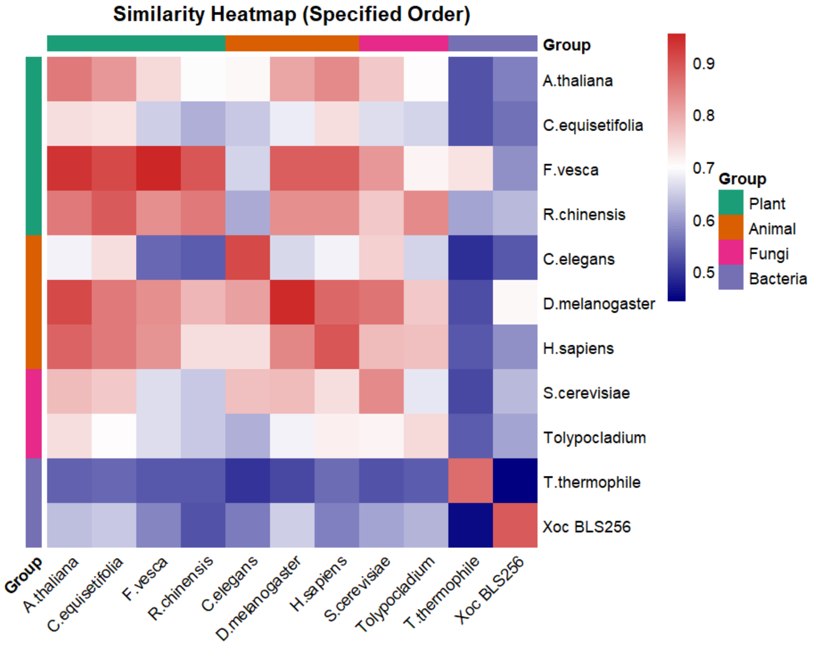

Fig. S5. A Cross-Species Heat Map of the ACC
